# Usage and Effectiveness of a Fully Automated, Open-Access, Spanish Web-Based Smoking Cessation Program: Randomized Controlled Trial

**DOI:** 10.2196/jmir.3091

**Published:** 2014-04-23

**Authors:** Guillermo Mañanes, Miguel A Vallejo

**Affiliations:** ^1^Faculty of PsychologyDepartment of Clinical PsychologyNational Distance Education University (UNED)MadridSpain

**Keywords:** smoking cessation, Internet, intervention studies

## Abstract

**Background:**

The Internet is an optimal setting to provide massive access to tobacco treatments. To evaluate open-access Web-based smoking cessation programs in a real-world setting, adherence and retention data should be taken into account as much as abstinence rate.

**Objective:**

The objective was to analyze the usage and effectiveness of a fully automated, open-access, Web-based smoking cessation program by comparing interactive versus noninteractive versions.

**Methods:**

Participants were randomly assigned either to the interactive or noninteractive version of the program, both with identical content divided into 4 interdependent modules. At baseline, we collected demographic, psychological, and smoking characteristics of the smokers self-enrolled in the Web-based program of Universidad Nacional de Educación a Distancia (National Distance Education University; UNED) in Madrid, Spain. The following questionnaires were administered: the anxiety and depression subscales from the Symptom Checklist-90-Revised, the 4-item Perceived Stress Scale, and the Heaviness of Smoking Index. At 3 months, we analyzed dropout rates, module completion, user satisfaction, follow-up response rate, and self-assessed smoking abstinence.

**Results:**

A total of 23,213 smokers were registered, 50.06% (11,620/23,213) women and 49.94% (11,593/23,213) men, with a mean age of 39.5 years (SD 10.3). Of these, 46.10% (10,701/23,213) were married and 34.43% (7992/23,213) were single, 46.03% (10,686/23,213) had university education, and 78.73% (18,275/23,213) were employed. Participants smoked an average of 19.4 cigarettes per day (SD 10.3). Of the 11,861 smokers randomly assigned to the interactive version, 2720 (22.93%) completed the first module, 1052 (8.87%) the second, 624 (5.26%) the third, and 355 (2.99%) the fourth. Completion data was not available for the noninteractive version (no way to record it automatically). The 3-month follow-up questionnaire was completed by 1085 of 23,213 enrolled smokers (4.67%). Among them, 406 (37.42%) self-reported not smoking. No difference between groups was found. Assuming missing respondents continued to smoke, the abstinence rate was 1.74% (406/23,213), in which 22,678 were missing respondents. Among follow-up respondents, completing the 4 modules of the intervention increased the chances of smoking cessation (OR 1.95, 95% CI 1.27-2.97, *P*<.001), as did smoking 30 minutes (OR 1.58, 95% CI 1.04-2.39, *P*=.003) or 1 hour after waking (OR 1.93, 95% CI 1.27-2.93, *P*<.001) compared to smoking within the first 5 minutes after waking.

**Conclusions:**

The findings suggest that the UNED Web-based smoking cessation program was very accessible, but a high level of attrition was confirmed. This could be related to the ease of enrollment, its free character, and the absence of direct contact with professionals. It is concluded that, in practice, the greater the accessibility to the program, the lower the adherence and retention. Professional support from health services and the payment of a reimbursable fee could prevent high rates of attrition.

## Introduction

The World Health Organization [1] states that tobacco consumption causes 5.6 million deaths through lung cancer, heart disease, stroke, and other diseases. This number reaches 6 million if passive smokers’ deaths are considered. Other data shows that in the European Union [2] 650,000 people die each year because of tobacco consumption, 443,000 deaths each year are attributable to tobacco use in the United States [3], and 55,000 people die every year from tobacco-related diseases in Spain [4,5].

Smokers are aware of the harmful effects of tobacco consumption: 70% would like to quit smoking and half try to quit each year, mostly without professional help [6-11]. Between 3% and 4% successfully quit smoking [12,13].

A problem of this magnitude requires treatments that are both effective and accessible to prevent millions of deaths worldwide [14-16], especially because treatments for tobacco dependence are available for only 14% of the world population [15]. Many smokers do not want to or cannot receive conventional treatment [17]. The Internet may be an effective, accessible, and efficient alternative in such cases [18-24].

Prior work has shown that interactive Web-based interventions for smoking cessation can be more effective than static websites and that there is a relationship between dose of intervention and its effect [23-31]. In relation to smokers’ characteristics that affect smoking cessation, some studies have indicated that a higher level of education and a lower number of cigarettes smoked are related to successful smoking cessation [23,32].

Concerning the usage of open-access eHealth programs, some studies have shown massive enrollment of users followed by a high level of dropout in the initial phase of treatment, without second visits to the website. The proportion of dropouts and nonusers decreases during treatment and subsequent follow-up in logarithmic progression. However, users who are registered in these open and public websites can benefit just as much from treatment as the participants in clinical studies, in which there is greater control over the recruitment of subjects and their behavior in the program [31,33-35]. Little is known about the users’ characteristics and the effectiveness and usage of fully automated Web-based interventions for smoking cessation in a real-world setting, without control or selection of users and provided free of charge. In Spain, no fully automated and open-access programs to quit smoking have been evaluated. The authors developed the Universidad Nacional de Educación a Distancia (National Distance Education University, UNED) Web-based program to offer an accessible alternative to millions of smokers who wish to quit smoking without attending conventional treatment sessions.

The aim of this paper is to describe the demographic, consumption, and psychological characteristics of 23,213 participants self-enrolled in the UNED Web-based smoking cessation program, to analyze the usage and effectiveness of the program, and the differences between 2 versions: an interactive automated control on the progress of the user and another without control or interactivity. We also examine if the participants’ adherence to treatment is related to the effectiveness of the intervention, and which user characteristics predict abstinence.

The hypotheses tested were (1) the interactive and tailored version of the program will yield higher quit rates than the static version, (2) usage of the modules of the intervention will drop drastically from the first module, (3) exposure to the content of the program will improve quit rates, and (4) the participants with higher education or lower physical dependence are more likely to achieve abstinence. Additionally, independent variables predicting module completion (ie, adherence to treatment) and follow-up response (ie, retention) were analyzed.

## Methods

### Ethical Approval

The study was a service open to all comers. The Bioethical Committee of the UNED approved the study. Registration of this trial was not required. Before starting the experiment, informed consent was obtained from the participants.

### Participants and Recruitment

Participants confirmed via the Web the following requirements before they could start the intervention: not undergoing other treatments to quit smoking, being older than 18 years, wishing to cease tobacco consumption in the next 30 days, smoking at least 2 cigarettes per day, having Internet access and an email address, and accepting the treatment conditions. Participants were informed that they had to complete a follow-up questionnaire 3 months after the beginning of the intervention.

At baseline, we collected demographic, psychological, and smoking characteristics data from the self-enrolled participants on the open-access UNED Web-based smoking cessation program [36] from October 2009 to May 2010, through a mandatory 61-item questionnaire. No direct contact with participants was made at any time. No economic incentives were employed. At the home page of the website, the participants were informed about the research nature of the program and about the researchers’ UNED affiliations. The launch of the program in October 2009 was announced by the university press office to mass media.

Two days after filling out the baseline questionnaire, the user received a link by email to register in the program. Once the link was activated, the smoker accessed a randomly assigned version of the program and the baseline questionnaire. The obligation to fill out the 61-item self-administered questionnaire and the 2-day delay in treatment access aimed to prevent the enrollment of impulsive smokers who had no intention of following the treatment.

At 3 months, participants were automatically reminded by email to complete a questionnaire about satisfaction with the program along with smoking and psychological variables. There was no live contact with the users to remind them to fill out the follow-up form.

### Measures

Participants self-reported their age, sex, nationality, marital status, education, and employment status. Psychological measurement instruments previously used online [26,37,38] were selected to avoid uncontrolled effects because of their use via Internet [39]. Psychological variables were assessed with the following questionnaires: the Symptom Checklist-90-Revised (SCL 90-R) [40], anxiety and depression subscales, and the 4-item Perceived Stress Scale (PSS-4) [41]. The PSS-4 is a reduced version of the 14-item PSS. It measures the degree to which the respondent has perceived stressful situations during the past month. Higher scores are correlated to more stress.

The participants’ self-reported on when they started smoking, physician’s advice on smoking cessation, motivation to quit smoking, living with smokers, and expectations of treatment success. Physical dependence on nicotine was measured with the Heaviness of Smoking Index (HSI) [42]. The HSI is a reduced 2-item version of the Fagerstrom Tolerance Questionnaire (FTQ) [43]. It measures nicotine dependence by using 2 questions from the FTQ: time of first cigarette in the morning and the number of cigarettes smoked per day. For each item, scores range from 0 to 3. The total score is the sum of the score on these 2 items. Nicotine dependence is then categorized into a 3-category variable: low (0-1), medium (2-4), and high (5-6). The HSI is used when time and resources are scarce [44].

To analyze utilization of the program, at 3 months we studied dropout rates, module completion, satisfaction with the program, and follow-up response rate. The participants could leave the program formally through the program menu option so that they were registered as a dropout. Module completion data was obtained automatically from the module questionnaire. Only when all the questions from each module were correctly answered was the smoker allowed to advance to the next module and module completion registered by the program. That information was only accessible from the interactive version. Satisfaction with the program was rated by the participants on a 5-level scale from 0=not at all satisfied to 4=extremely satisfied.

The follow-up response rate was calculated from the number of participants who completed the 3-month follow-up assessment. There was no contact by phone or by other means to reach the missing respondents.

The effectiveness of the program was measured by self-reported smoking status by using complete case and intention-to-treat analyses (ITT) at 3 months after registration. However, in cases of high dropout rates or very small follow-up response rate, ITT analysis could underestimate the effect of the program on the participants who were using it and who had been exposed to the intervention [34]. Because we were evaluating the effectiveness of an open-access Web-based intervention, exposure to and usage of the program is an essential condition to analyze its effect on abstinence. Otherwise, we could be assessing the follow-up success of the program instead of its effectiveness [45].

### Description of the Program

The content of the UNED Web-based smoking cessation program is divided into 4 consecutive modules. The intervention followed the Clinical Guidelines for the Treatment of Smoking [27] and is based on cognitive behavioral therapy methods tested effectively in conventional face-to-face smoking cessation programs [46,47]: education about the quit process, nicotine fading, self-monitoring, self-control, relapse prevention, coping skills, and lifestyle change. Two versions of the same content were implemented: interactive and noninteractive. For both versions, and according to specific algorithms, the output of the program depended on the users’ answers to the requirements of the intervention, acceptance of the treatment conditions, and completion of the pretreatment and posttreatment assessments. Both the scores of the psychological scales and the baseline questionnaire report were also automatically obtained through algorithms.

The program randomly assigned the users to either interactive or noninteractive versions of the program, both with the same therapeutic content. In the interactive format, the user had to follow a particular sequence of treatment in such a way that the modules were presented from first to fourth. Each module incorporated an evaluation form, which, if not answered correctly, prevented progress in the treatment sequence in order to guarantee that users had received the contents gradually, according to its own progress, and that users who had completed the last module had been exposed to all the content. An algorithm also allowed or prevented advancing to the next module depending on the time that had elapsed since the start of the module. In addition, another algorithm reminded the users through email that a week had passed and they had not completed the respective module. Greater exposure to the treatment was the aim. The previously completed modules were available to the user for review.

In the noninteractive version, users received identical content to that in the interactive version through a link to a single static Portable Document Format (PDF) file. Participants decided by themselves either to follow the normal sequence of modules or to skip some content. In this version, the program could control neither the behavior nor the progress of the user or the modules’ completion.

### Statistical Analysis

#### Overview

An alpha level of .05 was used for the statistical tests. Pearson correlation coefficient (*r*) was used to relate the quantitative variables and the chi-square test (χ^2^) for the categorical variables. The relation between categorical and quantitative variables was calculated with the Student *t* test.

#### Effect Size

Given the large sample size of the study, some very small differences could be statistically significant; therefore, we calculated the strength of relationship or effect size through Cohen’s *d* index for *t* test and Cramer’s V for chi-square test, according to Cohen’s criteria. For Cohen’s *d*, the small, medium, and large effect sizes are .20, .50, and .80, respectively. For Cramer’s V, the small, medium, and large effect sizes are calculated from the w index according to the number of categories in the variable. For the Pearson correlation coefficient (*r*), small, medium, and large effect sizes are .10, .30, and .50, respectively [48,49].

#### Statistical Power

With a small sample size, a lack of statistical significance does not necessarily mean the absence of differences, but rather low statistical power of the test. The statistical power depends on the number of subjects, the significance level, and the effect size. In our study, the large number of participants in the sample allowed us to detect small effect sizes in differences of means, correlations, and chi-square tests.

The sample size required to detect small effect sizes with a statistical power of alpha=.8 and an alpha=.5 is 393 for mean difference, 783 for Pearson’s *r*, and between 785 and 1362 subjects (depending on the degrees of freedom) in the case of the chi-square test. The sample size required to detect medium effect sizes with a statistical power of alpha=.8 and an alpha=.5 is 64 for mean difference, 85 for Pearson’s *r*, and between 87 and 151 subjects (depending on the degrees of freedom) in the case of the chi-square test [49].

#### Logistic Regression

Logistic regression analysis was used to examine the relationship between smoking cessation and predictor variables. Additionally, we examined completion of the program and the 3-month follow-up response as dependent variables. We calculated adjusted odds ratio (OR) for every predictor variable to understand its effect on the dependent variable. Following the recommendations of Hosmer and Lemeshow [50], the initial model incorporated predictor variables with significant value of less than .25 in the preliminary bivariate analysis using chi-square and Student *t* tests. The forward stepwise method was used to test variables for entry into the model one by one. After each entry, variables that were already in the model were tested for possible removal based on the significance of likelihood ratio.

## Results

### Overview

**Figure 1:**
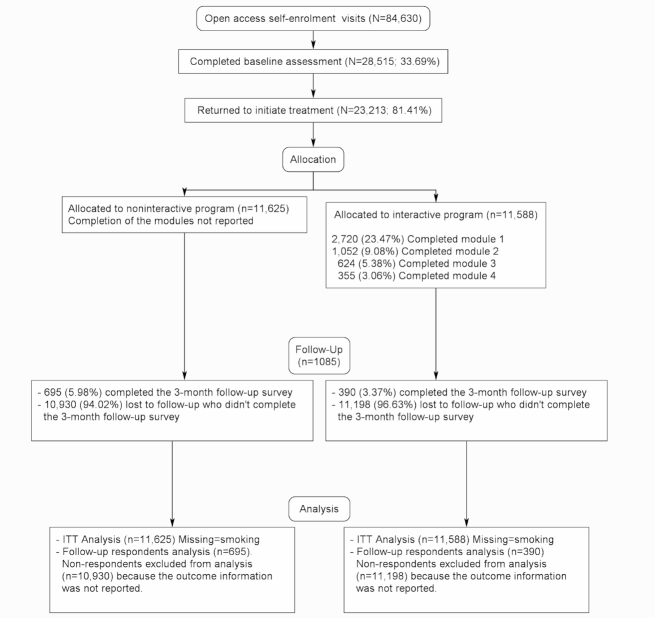
CONSORT flow diagram of visits to the website, participants who completed assessments, started treatment, and their allocation, as well as completers, follow-up, and analysis.

The Consolidated Standards of Reporting Trials (CONSORT) flow diagram with the number of participants randomly assigned to each group, participants who completed each module of the treatment in the interactive group, and participants analyzed for the outcome is shown in Figure 1.

### Participant Demographic Characteristics

Demographic characteristics at baseline are presented in Table 1. Randomization was successful, with neither group differing significantly on number of enrollees (11,902 in the noninteractive and 11,861 in the interactive, *P*=.79) or any baseline variable. The mean age of participants was 39.5 years (SD 10.3) and most were of Spanish nationality (93.57%, 21,721/23,213), married (46.10%, 10,701/23,213), with university education (46.03%, 10,686/23,213), and employed (78.73%, 18,275/23,213).

**Table 1:** Demographic characteristics of the program users.

Variable			Total	Version		*P*	Cramer’s V
				Noninteractive	Interactive		
Users registered, n (%)			23, 213 (100)	11,625 (50.07)	11,588 (49.93)	.79	
**Sex, n (%)**						.14	
		Male	11,593 (49.94)	5862 (50.42)	5731 (49.46)		
		Female	11,620 (50.06)	5763 (49.58)	5857 (50.54)		
Age, mean (SD)			39.50 (10.3)	39.56 (10.33)	39.43 (10.30)	.36	
	**Nationality, n (%)**					.47	
		Spanish	21,721 (93.57)	10,867 (93.48)	10,854 (93.66)		
		Other EU^a^	336 (1.48)	162 (1.39)	174 (1.50)		
		Non-EU^a^	1156 (4.98)	596 (5.13)	560 (4.84)		
	**Marital status, n (%)**					.62	
		Single	7992 (34.43)	4009 (34.48)	3983 (34.37)		
		Married	10,701 (46.10)	5328 (45.83)	5373 (46.36)		
		Separated	2099 (9.04)	1066 (9.16)	1033 (8.91)		
		Living as a couple	2192 (9.44)	1097 (9.44)	1095 (9.45)		
		Widowed	229 (0.99)	125 (1.09)	104 (0.91)		
	**Education, n (%)**					.54	
		Primary	3064 (13.20)	1502 (12.92)	1562 (13.48)		
		High school	5002 (21.55)	2535 (21.81)	2467 (21.29)		
		Professional training	4461 (19.22)	2242 (19.28)	2219 (19.15)		
		University	10,686 (46.03)	5346 (45.99)	5340 (46.08)		
	**Employment status, n (%)**					.07	0.012
		Working	18,275 (78.73)	9096 (78.24)	9179 (79.21)		
		Unemployed	4938 (21.27)	2529 (21.76)	2409 (20.79)		

^a^EU=European Union.

### Participant Smoking Variables

Program users’ mean daily tobacco consumption was 19.3 cigarettes (SD 10.3). Most participants smoked within the first half hour upon waking up. According to the HSI [44], nicotine dependence was medium in 48.16% (11,179/23,213) of participants and low in 34.85% (8090/23,213). Average age at onset of smoking was 17.3 years (SD 3.6). Of the participants, 64.27% (14,921/23,213) had received medical advice to quit smoking. Desire for abstinence was high (mean 7.9/10, SD 1.9), and expectations of success were positive (56.85%, 13,197/23,213). Both groups differed significantly on desire for abstinence and expectations of success, but with a very small effect size (Table 2).

Additional analysis showed that the number of cigarettes smoked per day increased in males, with age, with lower educational level, and in the case of separated people and widowers, with medium effect sizes for age and education and small effect sizes for sex and marital status (Table 3).

**Table 2:** Smoking characteristics of the program users.

Variable		Total	Version		*P*	Cohen’s *d*	Cramer’s V
			Noninteractive	Interactive			
Cigarettes per day, mean (SD)		19.3 (10.3)	19.4 (10.3)	19.3 (10.3)	.50		
**First cigarette of the day, n (%)**					.22		
	≤5 min	5431 (24.48)	2760 (50.81)	2671 (49.19)			
	6-30 min	9891 (44.59)	4987 (50.42)	4904 (49.58)			
	31-60 min	3687 (16.62)	1803 (48.90)	1884 (51.10)			
	>60 min	3171 (14.31)	1564 (52.16)	1607 (47.84)			
**HSI,** ^a^ **n (%)**					.32		
	Low	7647 (34.84)	3780 (49.43)	3867 (50.57)			
	Medium	10,569 (48.16)	5305 (50.19)	5264 (49.81)			
	High	3729 (17.00)	1897 (50.87)	1832 (49.13)			
Age at onset, mean (SD)		17.3 (3.6)	17.4 (3.7)	17.4 (3.6)	.73		
**Physician’s advice, n (%)**					.30		
	Yes	14,921 (64.27)	7510 (50.33)	7411 (49.67)			
	No	8292 (35.73)	4115 (49.62)	4177 (50.38)			
Desire for abstinence (0-10), mean (SD)		7.9 (1.9)	8.0 (1.9)	7.9 (2.0)	.01	.00	
**Lives with smokers, n (%)**					.94		
	Yes	14,267 (61.46)	7142 (50.05)	7125 (49.95)			
	No	8946 (38.54)	4483 (50.11)	4463 (49.89)			
**Expectations of success, n (%)**					.02		0.021
	Not at all	1030 (4.44)	488 (47.38)	542 (52.62)			
	Some	8986 (38.71)	4426 (49.25)	4560 (50.75)			
	Pretty much	9014 (38.83)	4553 (50.51)	4461 (49.94)			
	Completely	4183 (18.02)	2158 (51.59)	2025 (48.41)			

^a^HSI=Heaviness of Smoking Index.

**Table 3:** Users according to number of cigarettes smoked and demographic variables.

Variable		Number of cigarettes, %						*P*	Cramer’s V
		≤10 n=5026	11-20 n=11,688	21-30 n=4292	31-40 n=1799	41-50 n=267	≥51 n=139		
**Sex**								<.001	0.191
	Male	17.32	46.81	22.36	10.74	1.66	1.11		
	Female	26.08	53.94	14.51	4.81	0.56	0.10		
**Age**								<.001	0.150
	≤31	31.62	54.52	11.22	2.32	0.21	0.11		
	32-39	22.66	52.81	17.72	5.81	0.58	0.42		
	40-47	16.30	49.22	22.31	10.22	1.42	0.53		
	≥48	15.19	44.31	23.25	13.22	2.32	1.71		
**Education**								<.001	0.153
	Primary	11.97	49.01	24.02	11.78	2.22	1.00		
	High school	18.65	49.15	20.82	9.31	1.25	0.82		
	Professional training	20.71	52.52	18.10	7.81	0.72	0.41		
	University	26.15	50.42	16.11	5.74	0.92	0.66		
**Marital status**								<.001	0.076
	Single	27.42	52.04	14.86	4.72	0.68	0.28		
	Married	18.61	49.64	20.49	9.06	1.38	0.82		
	Separated	16.24	47.12	22.02	12.11	1.49	1.02		
	Living as a couple	21.81	50.75	18.12	7.74	1.01	0.57		
	Widowed	14.38	45.38	26.09	10.99	2.72	0.44		

### Participant Psychological Variables

Mean scores on anxiety, depression, and stress were not clinically significant. Groups did not differ significantly on any score. Additional analysis showed a significant relation between cigarette consumption and the psychological variables, but with a very small effect size (Table 4). The relationship between the depression subscale and the number of cigarettes smoked in men showed a small effect size (Cohen’s *d*=.11); in women, small effect sizes were observed in the depression subscale and the PSS-4 in relation to cigarettes smoked (Cohen’s *d*=.12 and .11, respectively).

**Table 4:** Pearson correlations between psychological variables and number of cigarettes smoked.

Scales	Anxiety^a^		Depression^b^		PSS^c^		Cigarettes	
	*r*	n	*r*	n	*r*	n	*r*	n
Anxiety^a^	-	23,212						
Depression^b^	.67^d^	23,212	-	23,213				
PSS-4^c^	.48^d^	23,212	.67^d^	23,213	-	23,213		
Cigarettes	.05^d^	22,885	.08^d^	22,886	.08^d^	22,886	-	22,886

^a^Anxiety subscale of the SCL-90-R.

^b^Depression subscale of the SCL-90-R.

^c^PSS-4: 4-item Perceived Stress Scale.

^d^ *P*<.001

### Dropout Rates and Module Completion

Of the 23,213 self-enrolled smokers, 1326 (5.71%) formally dropped out of the program. There were statistically significant differences between the interactive and noninteractive groups. The interactive group had higher dropout rates than the noninteractive (6.87%, 816/11861 vs 4.28%, 510/11902; χ^2^_1_=75.9, *P*<.001; Cramer’s V=0.057, *P*<.001), with very small effect size.

**Figure 2:**
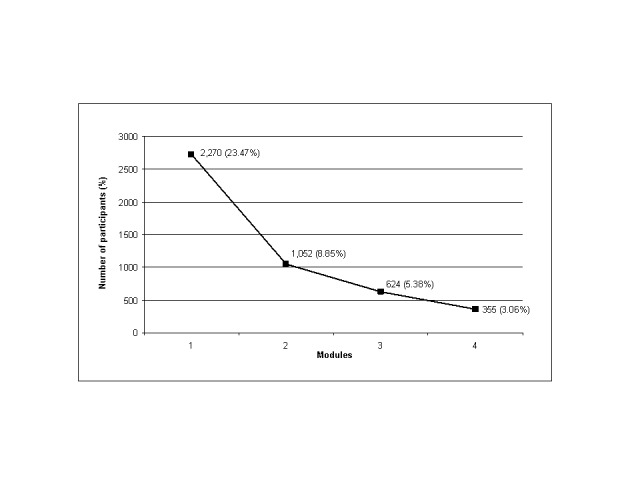
Attrition diagram of the modules completed against number of participants in the interactive group (n=11,588).

Of the 11,861 participants randomly assigned to the interactive version, 2720 (22.93%) completed the first module, 1052 (8.87%) the second, 624 (5.26%) the third, and 355 (2.99%) the fourth (Figure 2). Completion data was not available for the static PDF version because there was no way to record it automatically.

Logistic regression analyses revealed that being a Spaniard (OR 4.32, 95% CI 1.77-10.51, *P*<.001), aged between 40 and 47 years (OR 1.90, 95% CI 1.38-2.61, *P*<.001) or older than 48 years (OR 1.61, 95% CI 1.14-2.27, *P*=.01), having university education (OR 1.76, 95% CI 1.16-2.67, *P*=.01), and “some,” “pretty much,” and “complete” expectations of success (OR 3.72, 95% CI 1.17-11.79, *P*=.05; OR 6.03, 95% CI 1.91-18.98, *P*<.001; OR 5.49, 95% CI 1.72-17.57, *P*<.001, respectively) increased the chances of respondents completing all the intervention modules. Being male (OR 0.76, 95% CI 0.61-0.95, *P*=.05), smoking between 16 and 20 (OR 0.74, 95% CI 0.58-0.96, *P*=.05) or more than 20 cigarettes per day (OR 0.65, 95% CI 0.48-0.87, *P*<.001), and a higher level of stress (OR 0.94, 95% CI 0.90-0.98, *P*<.001) decreased chances (Table 5). There was no significant effect (95% CI for the OR includes 1) for the other variables included in the initial model, HSI and anxiety.

**Table 5:** Logistic regression predicting completion of the intervention in the interactive version (n=10,856).

Variable		B	SE	Wald	*df*	*P*	OR	95% CI
Sex (male)		–0.28	0.12	5.63	1	.02	0.76	0.61-0.95
**Age (years)**								
	≤31	Ref		21.28	3	<.001		
	32-39	0.11	0.18	0.37	1	.55	1.11	0.79-1.57
	40-47	0.64	0.16	15.60	1	<.001	1.90	1.38-2.61
	≥48	0.48	0.18	7.41	1	.01	1.61	1.14-2.27
Nationality (Spanish)		1.46	0.45	10.38	1	<.001	4.32	1.77-10.51
**Education**								
	Primary	Ref		8.20	2	.02		
	Secondary	0.37	0.22	2.90	1	.09	1.44	0.95-2.20
	University	0.57	0.21	7.12	1	.01	1.76	1.16-2.67
**Cigarettes per day**								
	≤15	Ref		9.83	2	.01		
	16-20	–0.30	0.13	5.14	1	.02	0.74	0.58-0.96
	≥21	–0.43	0.15	8.22	1	<.001	0.65	0.48-0.87
**Expectation of success**								
	Not at all	Ref		21.89	3	<.001		
	Some	1.31	0.59	4.99	1	.03	3.72	1.17-11.79
	Pretty much	1.80	0.59	9.42	1	<.001	6.03	1.91-18.98
	Completely	1.70	0.59	8.25	1	<.001	5.49	1.72-17.57
Stress		–0.07	0.02	10.10	1	<.001	0.94	0.90-0.98
Constant		–6.27	0.80	62.28	1	<.001	0.00	

### Follow-Up Response Rate

At 3 months, 1085 (4.67%) of the 23,213 smokers enrolled in the program completed a follow-up questionnaire about smoking status and satisfaction with the program. The 2 groups differed in the proportion of assessments: in the interactive group, 390 (3.29%) of 11,902 participants completed the follow-up questionnaire, whereas 695 of 11,861 (5.84%) did so in the noninteractive (χ^2^_1_=88.7, *P*=.01; Cramer’s V=0.061, *P*=.01) with very small effect size.

Logistic regression analyses showed that being Spanish (OR 2.70, 95% CI 1.82-4.01, *P*<.001); aged between 32 and 39 years (OR 1.27, 95% CI 1.05-1.54, *P*=.01), between 40 and 47 years (OR 1.52, 95% CI 1.26-1.83, *P*<.001), or older than 47 years (OR 1.57, 95% CI 1.29-1.91, *P*<.001); having a higher level of education (secondary level: OR 1.36, 95% CI 1.07-1.74, *P*=.01; university: OR 1.96, 95% CI 1.54-2.48, *P*<.001); smoking between 16 and 20 (OR 1.43, 95% CI 1.20-1.69, *P*<.001) or more than 20 cigarettes per day (OR 1.20, 95% CI 1.01-1.43, *P*=.04); and having “pretty much” (OR 1.91, 95% CI 1.27-2.88, *P*<.001) or “complete” expectations (OR 1.99, 95% CI 1.31-3.04, *P*<.001) of success in the and noninteractive group (OR 1.78, 95% CI 1.56-2.03, *P*<.001) increased the chances of completing the follow-up questionnaire. Being male decreased chances (OR 0.85, 95% CI 0.75-0.97, *P*=.02) (Table 6). There was no significant effect (95% CI for the OR includes 1) of the other variables included in the initial model: marital status, first cigarette of the day, and level of dependence on nicotine.

**Table 6:** Logistic regression predicting 3-month follow-up response (n=21,385).

Variable		B	SE	Wald	*df*	*P*	OR	95% CI
Sex (male)		–0.16	0.07	5.66	1	.02	0.85	0.75-0.97
**Age (years)**								
	≤31	Ref		25.97	3	<.001		
	32-39	0.24	0.10	6.21	1	.01	1.27	1.05-1.54
	40-47	0.42	0.10	19.09	1	<.001	1.52	1.26-1.83
	≥48	0.45	0.10	20.65	1	<.001	1.57	1.29-1.91
Nationality (Spanish)		0.99	0.20	24.07	1	<.001	2.70	1.82-4.01
**Education**								
	Primary	Ref		46.59	2	<.001		
	Secondary	0.31	0.13	6.10	1	.01	1.36	1.07-1.74
	University	0.67	0.12	30.51	1	<.001	1.96	1.54-2.48
**Cigarettes per day**								
	≤15	Ref		16.91	2	<.001		
	16-20	0.35	0.09	16.55	1	<.001	1.43	1.20-1.69
	≥21	0.19	0.09	4.37	1	.04	1.20	1.01-1.43
**Expectation of success**								
	Not at all	Ref		25.44	3	<.001		
	Some	0.37	0.21	3.01	1	.08	1.44	0.95-2.18
	Pretty much	0.65	0.21	9.57	1	<.001	1.91	1.27-2.88
	Complete	0.69	0.22	10.23	1	<.001	1.99	1.31-3.04
Version of the program (noninteractive)		0.58	0.07	73.91	1	<.001	1.78	1.56-2.03
Constant		–5.69	0.32	308.90	1	<.001	0.00	

### Satisfaction With the Program

User satisfaction was reported at 3-month follow-up (n=1085). The participants rated their satisfaction from not at all satisfied to extremely satisfied. Among them, 11.15% (121/1085) were not at all satisfied, 19.26% (209/1085) were slightly satisfied, 34.10% (370/1085) were somewhat satisfied, 25.81% (280/1085) were very satisfied, and 9.67% (105/1085) were extremely satisfied. There were differences in satisfaction in terms of the version of the program (χ^2^_4_=25.4, *P*<.001; Cramer’s V=0.153, *P*<.001) such that users of the interactive version showed higher proportions of very satisfied and extremely satisfied. Older people were more satisfied (χ^2^_12_=39.8, *P*<.001; Cramer’s V=0.111, *P*<.001), as were completers (χ^2^_4_=47.9, *P*<.001; Cramer’s V=0.350, *P*<.001), abstainers (χ^2^_4_=97.2, *P*<.001; Cramer’s V=0.299, *P*<.001), and participants with positive expectations (χ^2^_12_=73.1, *P*<.001; Cramer’s V=0.260, *P*<.001).

### Tobacco Cessation

At 3 months, 1085 users reported their smoking status. Because 97.69% (22,678/23,213) of the participants did not report outcome information, we conducted both ITT analysis and follow-up respondent analysis.

### Intention-to-Treat Analysis

Assuming the missing respondents continued to smoke, the abstinence rate was 1.74% (406/23,213), in which 22,678 were missing respondents. There was a significant difference in abstinence rates between groups (χ^2^_1_=26.7, *P*<.001; Cramer’s V=0.034, *P*<.001) with a very small effect size.

Logistic regression analyses revealed that being married or living as a couple (OR 1.26, 95% CI 1.02-1.55, *P*=.03) and complete expectations of success (OR 2.23, 95% CI 1.12-4.46, *P*=.02) increased the chances of quitting smoking. Smoking between 16 and 20 cigarettes per day (OR 0.70, 95% CI 0.56-0.89, *P*<.001) or more than 20 cigarettes per day (OR 0.55, 95% CI 0.42-0.72, *P*<.001), and using the interactive version of the program (OR 0.60, 95% CI 0.49-0.74, *P*<.001) decreased chances of quitting (Table 7). There was no significant effect of the other variables included in the initial model, time to first cigarette of the day, HSI, physician’s advice, and depression.

**Table 7:** Logistic regression predicting smoking cessation at 3 months (n=21,707).

Variable		B	SE	Wald	*df*	*P*	OR	95% CI
Marital status (married/couple)		0.23	0.11	4.70	1	.03	1.26	1.02-1.55
**Cigarettes per day**								
	≤15	Ref		21.15	2	<.001		
	16-20	–0.35	0.12	8.71	1	<.001	0.70	0.56-0.89
	≥21	–0.59	0.14	18.70	1	<.001	0.55	0.42-0.72
**Expectation of success**								
	Not at all	Ref		10.97	3	.01		
	Some	0.43	0.35	1.55	1	.21	1.54	0.78-3.04
	Pretty much	0.68	0.35	3.85	1	.05	1.97	1.00-3.86
	Complete	0.80	0.35	5.19	1	.02	2.23	1.12-4.46
Version of the program (interactive)		–0.51	0.11	22.55	1	<.001	0.60	0.49-0.74
Constant		–4.27	0.35	150.76	1	<.001	0.01	

### Follow-Up Respondent Analysis

Considering only the participants who responded to the follow-up questionnaire (n=1085), the abstinence rate was 37.42% (406/1085), whereas 62.58% (679/1085) self-reported as continuing to smoke. There was no significant difference in self-reported abstinence rates between groups (χ^2^_1_=0.4, *P*=.50; Cramer’s V=0.02, *P*=.50).

Logistic regression analyses revealed that smokers who had their first cigarette of the day after 30 minutes (OR 1.58, 95% CI 1.04-2.39, *P*=.003) or 1 hour (OR 1.93, 95% CI 1.27-2.93, *P*<.001) of waking increased their chances of quitting compared to those who had their first cigarette earlier. There was no significant effect of the other variables included in the regression model, version of the program, age, sex, first cigarette of the day, number of cigarettes smoked, level of dependence on nicotine, expectations, physician advice, depression, and marital status.

The abstinence rate among the participants who completed the intervention was 46.47% (165/355) versus 35.49% (3727/10,500) among noncompleters (*P*=.01). To study the effect of module completion on abstinence (exposure to treatment), we conducted a secondary analysis with the participants who answered the follow-up questionnaire in the interactive version (n=375). Only module completion had a significant effect on smoking cessation (OR 1.95, 95% CI 1.27-2.97, *P*<.001). There was no significant effect of other variables included in the regression model, age, sex, first cigarette of the day, number of cigarettes smoked, level of dependence on nicotine, expectations, physician advice, depression, and marital status.

### Continuing to Smoke Analysis

Participants who self-reported they continued to smoke (676/23,213, 2.91%) significantly reduced both the average number of cigarettes smoked daily (from 17.9 to14) and the level of physical dependence on nicotine with medium effect sizes of .45 and .53, respectively (Table 8).

**Table 8:** Number of cigarettes smoked (n=628) and level of nicotine dependence (n=614) at baseline and after 3 months among participants who self-reported as continuing to smoke.

Variables	At baseline, mean (SD)	At 3 months, mean (SD)	Diff	*r*	*P*	*t (df)*	*P*	Cohen’s *d*
Cigarettes	17.90 (9.83)	14.00 (8.17)	3.90	.55	<.001	11.31 (627)	<.001	.45
HSI^a^	2.84 (1.54)	2.20 (1.51)	0.64	.68	<.001	13.04 (613)	<.001	.53

^a^ HSI=Heaviness of Smoking Index.

## Discussion

### Principal Findings

The aim of this paper was to describe the characteristics of participants self-enrolled in the UNED Web-based smoking cessation program and to analyze the program’s usage and effectiveness and the differences between its 2 versions: interactive and noninteractive. We also examined if participants’ exposure to treatment was related to its effectiveness and which user characteristics predicted abstinence. Additionally, independent variables predicting module completion (ie, adherence to treatment and follow-up response, such as retention) were analyzed.

From October 2009 to May 2010, 23,213 users self-enrolled in the UNED Web-based smoking cessation program, which was free of charge and fully automated. There were no differences in the demographic, psychological, and smoking characteristics between the 2 versions of the program.

### Participant Characteristics

The demographic and smoking characteristics of the study participants are similar to those reported in other Internet smoking cessation programs [23,32,51-55] except for sex: in the UNED program, the number of males and females was not significantly different, whereas in most other studies, females were the majority. This may be because the sample sizes were smaller in the mentioned studies, ranging from 351 [55] to 17,159 participants [51]. With larger samples, as in the case of Barrera and colleagues [51], the proportions of men and women were almost the same (49.3% men vs 50.7% women). In contrast, in the UNED, men’s participation among non-European users was almost twice that of women’s. Except for Spaniards, we did not register the nationality of each user, but rather European or non-European nationality. Because the program was provided only in the Spanish language and announced through national and regional mass media, we can assume that non-European users (n=1156) were primarily people from Latin American countries living in Spain. Despite the fact that there has been an increase in female tobacco consumption in recent years in Latin American countries, the proportion of male smokers is still triple or quadruple that of women in countries such as Ecuador, El Salvador, Guatemala, Honduras, Mexico, Nicaragua, Paraguay, and Peru [56].

The proportion of users with university education (46%) versus other categories is noteworthy, confirming once again the existence of a digital divide in the specific field of eHealth. In Spain, the percentage of people with higher education in 2009 was 16.29% versus 20.22% with primary education. However, that same year, the percentage of Internet users with higher education was 28.34% versus 7.85% with primary education according to the Instituto Nacional de Estadística (National Institute of Statistics) [57,58]. Therefore, we can confirm that smoking cessation programs are similar to other eHealth programs in that individuals with a lower educational level are underrepresented [59,60]. Moreover, a higher ability to understand, organize, and analyze written information is required for the use of Web-based programs compared to a traditional face-to-face intervention [20,61]. All this has a dissuasive effect on participants with a lower educational level. It has also been suggested that the excessive length of the written material employed in self-help formats may impede their use, especially by smokers who are not accustomed to dealing with this type of information [62].

Concerning scores of psychological scales, the results do not differ from those obtained in other studies in the general population [37,63]. In spite of the fact that cigarette consumption has been associated with higher levels of anxiety, depression, and stress [64,65], the correlation was nonsignificant in the present study.

### Usage of the Program

The registration of smokers in the program was high, with 23,213 participants self-enrolled in 8 months. This may be because of the characteristics of Web-based programs, which do not require transportation or schedules and, in the case of the UNED, were free of charge and open-access. In addition, the university press office announced the launch of the program to the mass media.

The number of users who completed the 4 modules in the program confirmed the law of attrition proposed by Eysenbach [34], according to which a high rate of noncompleters and those lost to follow-up are a typical feature of Web-based programs. Adherence to the intervention decreased dramatically over time: 22.93% of users completed the first module, 8.87% the second, 5.26% the third, and 2.99% the fourth. Despite the high motivation of the users and their positive expectations at baseline, we found only 2.99% of participants completed the intervention and 4.57% completed the 3-month follow-up even with the 2 measures implemented to prevent impulsive enrollment (ie, delayed access to the program and a long mandatory baseline questionnaire). These results are similar to those shown in other eHealth programs [33,35,66]. In the case of smoking cessation programs, comparison of adherence results between studies is difficult because either the usage measures employed do not include the proportion of users who complete the interventions or they are not structured into successive modules or sessions. For example, Etter [26] reported that 16% of users completed at least 2 of 3 interactive surveys of an intervention that consisted of providing therapeutic content according to the answers to each survey. McKay et al [32] reported an average of 1 visit to the website in a cognitive behavioral program. Seidman et al [23] reported an average of 8 completed sessions of a 32-session cognitive behavioral program, and Rabius et al [53] confirmed no more than 2 visits in a study with random allocation to 5 different websites to quit smoking. Although the unit of adherence measure differs between studies, there is a general underutilization of Web-based interventions, especially in open-access programs. Our hypothesis predicting a high rate of nonusers from the first module was confirmed.

Because a high number of participants enrolled over the course of 8 months (23,213), the UNED Web-based smoking cessation program was shown to be very accessible. No extra measures were used, except email reminders, to persuade the participants to continue using the program and respond to the follow-up questionnaire. The study confirmed that the easier it is to enroll or to leave the program, the higher the nonusage rate and the rate of those lost to follow-up will be, as Eysenbach [34] proposed.

In addition, the regression model showed that the strongest predictors of completing the program were positive expectations of success and being older than 40 years. This could be explained by older participants having an increased perception of the harmful effects of smoking on health compared to younger people, and by positive expectations being related to a high level of self-efficacy, both causing greater use of the application. Similar results were obtained by Zbikowski et al [31] and Wangberg et al [67].

### Effectiveness of the Program

The ITT abstinence rate was 1.71%. This result is difficult to interpret. ITT analysis is feasible when we know the outcome of every participant, regardless of whether they have completed the program or not. If the lack of data on the outcome is very high, ITT analysis is not feasible because considering every participant lost to follow-up as one without successful treatment will produce results that rely on unverifiable assumptions [68,69]. That the missing respondents continue to smoke is an untestable assumption about the missing data for 95.44% of the participants in the study, and this could bias results about the real effectiveness of the program [45]. Furthermore, Tomson et al [69] found that nonresponders to a Quitline trial were even more likely to be abstinent than responders.

If we consider the data from the participants who actually reported their smoking status at follow-up (ie, those we know the outcome), the cessation rate was 37.42%. This result is in the range of other studies that analyzed cessation rates in participants who answered a 3-month follow-up by phone or through the Web. Cobb et al [70] found a 44.6% abstinence rate and Etter [52] reported 37.3% at 11 weeks. In the study by McKay et al [32], the proportion was considerably lower, at 19.7%.

Despite the results of previous work [23,24,27,29,30,52] and in contrast to our first hypothesis, in the follow-up respondent analysis we found no differences between the 2 versions of the program—interactive and noninteractive—whereas in the ITT analysis the interactive version decreased chances of quitting. In the present study, both groups were exposed to the same content. The only difference was interactivity; consequently, the comparison was strictly interactivity versus no interactivity rather than interactivity versus another different intervention. To our knowledge, this is the first study that evaluated interactivity versus noninteractivity while maintaining the same content in both groups. However, we should confirm whether the absence of differences found in the study is maintained in the long term.

We consider the interactivity because this is the main experimental manipulation. Nevertheless, the interactive version includes several components: the regular programmed progression across the modules, the evaluation at the end of each module, and the necessity of interaction with the Web application to follow the programmed steps. Knowing the effect of the interactivity will be necessary to explore these variables.

The regression analysis showed that completion of the treatment (ie, exposure to the intervention) increased the chances of smoking cessation, which confirms our third hypothesis. However, contrary to our fourth hypothesis, we could not confirm the effect of education and physical dependence on smoking abstinence. There was no significant effect of any demographic variables on abstinence. Education affected the adherence to the program, but did not improve smoking cessation. Anxiety, depression, and stress did not affect abstinence either. This result is consistent with prior studies that have failed to demonstrate the effect of incorporating mood management components in the intervention [24,71].

### Limitations

First, the information obtained at baseline was not confirmed by other means. Second, self-reported abstinence was not validated chemically. In a program with more than 23,000 participants from any part of the world, data verification is not viable. Some studies confirmed the validity of self-reported abstinence when compared with chemical verification, becoming the standard method in Web-based studies [27,72,73]. Another limitation of the study is related to the 3-month follow-up. This period is not long enough to establish clear conclusions about the effectiveness of the program.

### Conclusions

The large number of enrollees in the program is encouraging in terms of accessibility and confirms the feasibility of delivering Web-based programs for smoking cessation to smokers that do not have access to conventional face-to-face programs. It would be very difficult to reach 23,213 smokers through a face-to-face intervention setting. This study showed that completion of the program (ie, adherence) improves the chances of cessation. However, high rates of nonusage and loss to follow-up were confirmed. To increase the program’s usage and follow-up completion, some professional involvement could be implemented in such a way as to ensure a self-obligation toward both the program and the professional [74]. For example, professional support from primary health care could prevent early dropout and reinforce adherence. It has been found that users of a Web-based smoking cessation program referred by general practitioners showed lower dropout rates compared to those who accessed the program directly [75]. Another therapeutic measure for increasing adherence to free-of-charge programs could be the payment of a fee to achieve higher commitment to the program, which would be reimbursable if completion is achieved.

To summarize, the main findings of this research are to disconfirm the general belief that anxiety, depression, and stress are associated with cigarette consumption [64,65] and confirmation that it is not necessary to incorporate mood management components in an intervention [24,71]. This outcome allows for configuration of an intervention less associated with clinical terms and more related to health behaviors. Secondly, the utility of a simple noninteractive intervention program versus a more complex interactive one. Finally, this study has shown the potential efficacy of an intervention for people with diverse levels of physical dependence and different education levels.

The results of our study suggest that future research is needed to determine (1) the factors that will reduce the high rates of attrition in public Web-based programs for smoking cessation, (2) the real proportion of nonsmokers among those lost to follow-up, and (3) the long-term abstinence rate in the UNED Web-based smoking cessation program.

## Abbreviations

CONSORT: Consolidated Standards of Reporting Trials
FTQ: Fagerstrom Tolerance Questionnaire
HSI: Heaviness of Smoking Index
ITT: intention-to-treat
PDF: Portable Document Format
PSS-4: 4-item Perceived Stress Scale
SCL 90-R: Symptom Checklist-90-Revised
UNED: Universidad Nacional de Educación a Distancia (National Distance Education University)

## References

[1] (2011). WHO report on the global tobacco epidemic, 2011: Warning about the dangers of tobacco.

[2] Commission of the European Communities (2007). Green paper Towards a Europe free from tobacco smoke: Policy options at EU level.

[3] Centers for Disease ControlPrevention (CDC) (2008). Smoking-attributable mortality, years of potential life lost, and productivity losses--United States, 2000-2004. MMWR Morb Mortal Wkly Rep.

[4] Banegas JR, Díez L, González J, Villar F, Rodriguez-Artalejo F (2005). La mortalidad atribuible al tabaquismo comienza a descender en España. Med Clin (Barc).

[5] Montes A, Pérez M, Gestal JJ (2004). Impacto del tabaquismo sobre la mortalidad en España. Adicciones.

[6] Centers for Disease ControlPrevention (CDC) (2011). Quitting smoking among adults--United States, 2001-2010. MMWR Morb Mortal Wkly Rep.

[7] Fiore MC, Novotny TE, Pierce JP, Giovino GA, Hatziandreu EJ, Newcomb PA, Surawicz TS, Davis RM (1990). Methods used to quit smoking in the United States. Do cessation programs help?. JAMA.

[8] Friend K, Levy DT (2002). Reductions in smoking prevalence and cigarette consumption associated with mass-media campaigns. Health Educ Res.

[9] Shiffman S, Brockwell SE, Pillitteri JL, Gitchell JG (2008). Use of smoking-cessation treatments in the United States. Am J Prev Med.

[10] Taylor T, Lader D, Bryant A, Keyse L, Joloza MT (2006). Office for National Statistics.

[11] Zhu S, Melcer T, Sun J, Rosbrook B, Pierce JP (2000). Smoking cessation with and without assistance: a population-based analysis. Am J Prev Med.

[12] Hughes JR, Keely J, Naud S (2004). Shape of the relapse curve and long-term abstinence among untreated smokers. Addiction.

[13] West R (2006). Smoking in England.

[14] Fiore MC, Croyle RT, Curry SJ, Cutler CM, Davis RM, Gordon C, Healton C, Koh HK, Orleans CT, Richling D, Satcher D, Seffrin J, Williams C, Williams LN, Keller PA, Baker TB (2004). Preventing 3 million premature deaths and helping 5 million smokers quit: a national action plan for tobacco cessation. Am J Public Health.

[15] (2011). World Health Organization.

[16] Task Force on Community Preventive Services (2001). Recommendations regarding interventions to reduce tobacco use and exposure to environmental tobacco smoke. Am J Prev Med.

[17] Curry SJ (2001). ASPO Joseph W. Cullen Memorial Award Lecture. Bridging the clinical and public health perspectives in tobacco treatment research: scenes from a tobacco treatment research career. Cancer Epidemiol Biomarkers Prev.

[18] Bennett GG, Glasgow RE (2009). The delivery of public health interventions via the Internet: actualizing their potential. Annu Rev Public Health.

[19] Civljak M, Sheikh A, Stead LF, Car J (2010). Internet-based interventions for smoking cessation. Cochrane Database Syst Rev.

[20] Etter JF (2002). Using new information technology to treat tobacco dependence. Respiration.

[21] Lancaster T, Stead LF (2005). Self-help interventions for smoking cessation. Cochrane Database Syst Rev.

[22] Myung SK, McDonnell DD, Kazinets G, Seo HG, Moskowitz JM (2009). Effects of Web- and computer-based smoking cessation programs: meta-analysis of randomized controlled trials. Arch Intern Med.

[23] Seidman DF, Westmaas JL, Goldband S, Rabius V, Katkin ES, Pike KJ, Wiatrek D, Sloan RP (2010). Randomized controlled trial of an interactive internet smoking cessation program with long-term follow-up. Ann Behav Med.

[24] Shahab L, McEwen A (2009). Online support for smoking cessation: a systematic review of the literature. Addiction.

[25] Cobb NK, Graham AL, Bock BC, Papandonatos G, Abrams DB (2005). Initial evaluation of a real-world Internet smoking cessation system. Nicotine Tob Res.

[26] Etter JF (2005). Comparing the efficacy of two Internet-based, computer-tailored smoking cessation programs: a randomized trial. J Med Internet Res.

[27] Fiore MC, Jaen CR, Baker TB, Bailey WC, Benowitz NL, Curry SJ (2008). Treating tobacco use and dependence: 2008 Update.

[28] Japuntich SJ, Zehner ME, Smith SS, Jorenby DE, Valdez JA, Fiore MC, Baker TB, Gustafson DH (2006). Smoking cessation via the internet: a randomized clinical trial of an internet intervention as adjuvant treatment in a smoking cessation intervention. Nicotine Tob Res.

[29] Lenert L, Muñoz RF, Perez JE, Bansod A (2004). Automated e-mail messaging as a tool for improving quit rates in an internet smoking cessation intervention. J Am Med Inform Assoc.

[30] Strecher VJ, Shiffman S, West R (2005). Randomized controlled trial of a web-based computer-tailored smoking cessation program as a supplement to nicotine patch therapy. Addiction.

[31] Zbikowski SM, Jack LM, McClure JB, Deprey M, Javitz HS, McAfee TA, Catz SL, Richards J, Bush T, Swan GE (2011). Utilization of services in a randomized trial testing phone- and web-based interventions for smoking cessation. Nicotine Tob Res.

[32] McKay HG, Danaher BG, Seeley JR, Lichtenstein E, Gau JM (2008). Comparing two web-based smoking cessation programs: randomized controlled trial. J Med Internet Res.

[33] Christensen H, Griffiths KM, Korten AE, Brittliffe K, Groves C (2004). A comparison of changes in anxiety and depression symptoms of spontaneous users and trial participants of a cognitive behavior therapy website. J Med Internet Res.

[34] Eysenbach G (2005). The law of attrition. J Med Internet Res.

[35] Farvolden P, Denisoff E, Selby P, Bagby RM, Rudy L (2005). Usage and longitudinal effectiveness of a Web-based self-help cognitive behavioral therapy program for panic disorder. J Med Internet Res.

[36] Dejar de Fumar.

[37] Herrero J, Meneses J (2006). Short web-based versions of the Perceived Stress (PSS) and Center for Epidemiological Studies - Depression (CESD) scales: A comparison to pencil and paper responses among Internet users. Comput Human Behav.

[38] Vallejo MA, Mañanes G, Isabel Comeche MA, Díaz MI (2008). Comparison between administration via Internet and paper-and-pencil administration of two clinical instruments: SCL-90-R and GHQ-28. J Behav Ther Exp Psychiatry.

[39] Buchanan T (2003). Internet-based questionnaire assessment: appropriate use in clinical contexts. Cogn Behav Ther.

[40] Derogatis LR (1977). SCL-90-R: Administration, scoring and procedures Manual I for the revised version of the SCL-90.

[41] Cohen S, Kamarck T, Mermelstein R (1983). A global measure of perceived stress. J Health Soc Behav.

[42] Heatherton TF, Kozlowski LT, Frecker RC, Rickert W, Robinson J (1989). Measuring the heaviness of smoking: using self-reported time to the first cigarette of the day and number of cigarettes smoked per day. Br J Addict.

[43] Fagerström KO (1978). Measuring degree of physical dependence to tobacco smoking with reference to individualization of treatment. Addict Behav.

[44] Heatherton TF, Kozlowski LT, Frecker RC, Fagerström KO (1991). The Fagerström Test for Nicotine Dependence: a revision of the Fagerström Tolerance Questionnaire. Br J Addict.

[45] Leykin Y, Aguilera A, Torres LD, Pérez-Stable EJ, Muñoz RF (2012). Interpreting the outcomes of automated internet-based randomized trials: example of an International Smoking Cessation Study. J Med Internet Res.

[46] Becoña E, Vázquez FL (1998). Tratamiento del tabaquismo.

[47] Abrams DB, Niaura R, Brown RA, Emmons KM, Goldestein MG, Monti PM (2003). The Tobacco Dependence Treatment Handbook: A Guide to Best Practices.

[48] Cohen J (1992). A power primer. Psychol Bull.

[49] Cohen J (1988). Statistical Power Analysis for the Behavioral Sciences, 2nd ed.

[50] Hosmer DW, Lemeshow S (2000). Applied Logistic Regression, 2nd ed.

[51] Barrera AZ, Pérez-Stable EJ, Delucchi KL, Muñoz RF (2009). Global reach of an Internet smoking cessation intervention among Spanish- and English-speaking smokers from 157 countries. Int J Environ Res Public Health.

[52] Etter JF (2005). Comparing the efficacy of two Internet-based, computer-tailored smoking cessation programs: a randomized trial. J Med Internet Res.

[53] Rabius V, Pike KJ, Wiatrek D, McAlister AL (2008). Comparing internet assistance for smoking cessation: 13-month follow-up of a six-arm randomized controlled trial. J Med Internet Res.

[54] Saul JE, Schillo BA, Evered S, Luxenberg MG, Kavanaugh A, Cobb N, An LC (2007). Impact of a statewide Internet-based tobacco cessation intervention. J Med Internet Res.

[55] Swartz LH, Noell JW, Schroeder SW, Ary DV (2006). A randomised control study of a fully automated internet based smoking cessation programme. Tob Control.

[56] Müller F, Wehbe L (2008). Smoking and smoking cessation in Latin America: a review of the current situation and available treatments. Int J Chron Obstruct Pulmon Dis.

[57] (2009). Instituto Nacional de Estadística.

[58] (2011). Instituto Nacional de Estadística.

[59] Neter E, Brainin E (2012). eHealth literacy: extending the digital divide to the realm of health information. J Med Internet Res.

[60] Norman CD, Skinner HA (2006). eHealth Literacy: Essential Skills for Consumer Health in a Networked World. J Med Internet Res.

[61] Meade CD, Byrd JC, Lee M (1989). Improving patient comprehension of literature on smoking. Am J Public Health.

[62] Strecher VJ, Kreuter M, Den Boer DJ, Kobrin S, Hospers HJ, Skinner CS (1994). The effects of computer-tailored smoking cessation messages in family practice settings. J Fam Pract.

[63] González-Rivera JL, De-las-Cuevas C, Rodríguez-Abuin M (2002). El cuestionario de 90 síntomas. Adaptación española del SCL-90-R [The 90-symptoms questionnaire. Spanish adaptation].

[64] Jamal M, Willem Van der Does AJ, Cuijpers P, Penninx BW (2012). Association of smoking and nicotine dependence with severity and course of symptoms in patients with depressive or anxiety disorder. Drug Alcohol Depend.

[65] Jorm AF, Rodgers B, Jacomb PA, Christensen H, Henderson S, Korten AE (1999). Smoking and mental health: results from a community survey. Med J Aust.

[66] Verheijden MW, Jans MP, Hildebrandt VH, Hopman-Rock M (2007). Rates and determinants of repeated participation in a web-based behavior change program for healthy body weight and healthy lifestyle. J Med Internet Res.

[67] Wangberg SC, Bergmo TS, Johnsen JA (2008). Adherence in Internet-based interventions. Patient Prefer Adherence.

[68] Lewis JA, Machin D (1993). Intention to treat--who should use ITT?. Br J Cancer.

[69] Tomson T, Björnström C, Gilljam H, Helgason A (2005). Are non-responders in a quitline evaluation more likely to be smokers?. BMC Public Health.

[70] Cobb NK, Graham AL, Bock BC, Papandonatos G, Abrams DB (2005). Initial evaluation of a real-world Internet smoking cessation system. Nicotine Tob Res.

[71] Muñoz RF, Barrera AZ, Delucchi K, Penilla C, Torres LD, Pérez-Stable EJ (2009). International Spanish/English Internet smoking cessation trial yields 20% abstinence rates at 1 year. Nicotine Tob Res.

[72] Patrick DL, Cheadle A, Thompson DC, Diehr P, Koepsell T, Kinne S (1994). The validity of self-reported smoking: a review and meta-analysis. Am J Public Health.

[73] Barrueco M, Jiménez Ruiz C, Palomo L, Torrecilla M, Romero P, Riesco JA (2005). Veracidad de la respuesta de los fumadores sobre su abstinencia en las consultas de deshabituación tabáquica. Archivos de Bronconeumología.

[74] Mohr DC, Cuijpers P, Lehman K (2011). Supportive accountability: a model for providing human support to enhance adherence to eHealth interventions. J Med Internet Res.

[75] Smit ES, Hoving C, Cox VC, de Vries H (2012). Influence of recruitment strategy on the reach and effect of a web-based multiple tailored smoking cessation intervention among Dutch adult smokers. Health Educ Res.

